# Enhancing HIV treatment and support: a qualitative inquiry into client and healthcare provider perspectives on differential service delivery models in Uganda

**DOI:** 10.1186/s12981-024-00637-0

**Published:** 2024-07-27

**Authors:** Simon Peter Katongole, Semei Christopher Mukama, Jane Nakawesi, Dedrix Bindeeba, Ezajob Simons, Andrew Mugisa, Catherine Senyimba, Eve Namitala, Robert Anguyo D. D. M. Onzima, Barbara Mukasa

**Affiliations:** 1 Mildmay, Kampala, Uganda; 2 Gudie University Project, P.O. Box 27450, Kampala, Uganda; 3 Liverpool School of Tropical Medicine (LSTM), Department of International Public Health, Liverpool, UK

## Abstract

**Background:**

HIV/AIDS continues to be a significant contributor to illness and death, particularly in sub-Saharan Africa. In this study, we conducted a qualitative assessment to understand Client and Healthcare Provider Perspectives on Differential Service Delivery Models in Uganda. The purpose was to establish strengths and weaknesses within the services delivery models, inform policy and decision-making, and to facilitate context specific solutions.

**Methods:**

Between February and April 2023, a qualitative cross-sectional study was utilised to gather insights from a targeted selection of individuals, including People Living with HIV (PLHIV), healthcare workers, HIV focal persons, community retail pharmacists, and various stakeholders. The data collection process included eleven in-depth interviews, nine key informant interviews, and eight focus group discussions carried out across eight districts in Central Uganda. The collected data was analyzed through inductive thematic analysis with the aid of Excel.

**Results:**

The various Differentiated Service Delivery Models (DSDMs), notably Community-Client-Led Drug Distribution (CCLAD), Community Drug Distribution Point (CDDP), Community Retail Pharmacy Drug Distribution Point (CRPDDP), and the facility-based Facility Based Individual Model (FBIM), were reported to have several positive impacts. These included improved treatment adherence, efficient management of antiretroviral (ARV) supplies, reduced exposure to infectious diseases, enhanced healthcare worker hospitality, minimized travel time for ART refills, stigma reduction, and decreased waiting times. Concern was raised about the lack of improvement in HIV status disclosure, opportunistic infection treatment, adherence to seasonal appointments, and sustainability due to the overreliance of the DSDMs on donor funding, suggesting potential discontinuation without funding. Doubts about health workers’ commitment surfaced. Notably, the CCLAD model displayed self-sustainability, with clients financially supporting group members to collect medicines.

**Conclusion:**

Community-based DSDMs, such as CCLAD and CDDP, improve ART refill convenience, social support, and client experiences. These models reduce travel and waiting times, lowering infection risks. Addressing challenges and enhancing facility-based models is vital. In order to maintain funding after donor funding ends, sustainability measures like cross-subsidization can be used. If well implemented, the DSDMs have the potential to produce better or comparable ART outcomes compared to the FBIM model.

**Supplementary Information:**

The online version contains supplementary material available at 10.1186/s12981-024-00637-0.

## Background

Ensuring effective and accessible HIV antiretroviral therapy (ART) remains a global health priority, particularly in resource-limited contexts [1]. Differential Service Delivery Models (DSDMs) have emerged as innovative strategies for optimizing ART access and retention, catering to the diverse needs of people living with HIV (PLHIV). In Uganda, where HIV prevalence is high, DSDMs play a vital role in widespread ART provision and enhancing care quality [2].

Five HIV DSDMs were introduced in Uganda in 2017 to strengthen ART access, reduce care costs, improve satisfaction, and boost ART outcomes for PLHIV [3]. These models include Community-Client-Led Drug Distribution (CCLAD), Community Drug Distribution Point (CDDP), Community Retail Pharmacy Drug Distribution Point (CRPDDP), Fast-Track Drug Refill (FTDR), and Facility-Based Individual Management (FBIM) [4]. In the DSDM context, standard health facility care is called Facility Based Individual Management (FBIM). In the CCLAD model, PLHIV form community-based groups of not more than six people thus rotating drug collection for group members once every three months. In CDDP, stable clients collect drugs from community fixed drug distribution points, and in the FTDR model, PLHIVs pick up pre-packed drugs from health facilities). The clients are educated to make informed choices before being placed in a particular model. However, unstable or non-adherent PLHIV are retained or re-enrolled in FBIM [5].

Uganda also supports HIV/AIDS social support strategies like Young People and Adolescent Peer Supporters (YAPS) and Caregiver’s DOTS for adolescents and children [6]. YAPS peers support HIV testing and partner notification among sexually active adolescents through peer-led index testing and counseling [7]. Meanwhile, the Caregiver-led DOTS model ensures a designated caregiver oversees ART administration for children aged 0–15 years with adherence challenges, promoting increased testing rates and improved health outcomes [7, 8].

Despite existing research on DSDM outcomes in HIV care, a significant gap remains in understanding the perceptions of both service providers and clients regarding service delivery within these models [9, 10]. Previous studies have primarily focused on identifying obstacles to service provision, neglecting a deeper analysis of the entire service delivery process [11, 12]. This oversight hinders the development of tailored interventions addressing the diverse needs of HIV-positive individuals [13–15]. To address this gap, further research is necessary to explore the perceptions and experiences of service providers and clients, enabling the creation of effective, patient-centered interventions that improve treatment adherence and health outcomes.

This study conducts an in-depth exploratory analysis to investigate the perspectives and experiences of healthcare practitioners, clients, and caregivers regarding DSDMs and client support groups. By understanding the challenges and efficacy of both DSDMs and support groups, healthcare professionals and policymakers can develop targeted interventions that cater to the unique needs of diverse subgroups within the HIV-positive population. The findings of this research have the potential to inform enhancements in HIV/AIDS programmatic approaches, ultimately improving the well-being of People Living with HIV (PLHIV).

## Methodology

### Study design

A qualitative, cross-sectional, descriptive, phenomenological approach was employed to conduct semi-structured in-depth interviews. In this descriptive phenomenology study, the primary objective was to precisely describe the perspectives, experiences, and perceptions of study participants about the DSDM and YAPS initiatives, without necessarily exploring in-depth interpretation or explanation.

### Study setting

This study was conducted between February and April 2023, across eight districts in central Uganda that have implemented Differential Service Delivery Models (DSDMs). The districts included Kiboga, Kyankwanzi, Mubende, Kassanda, Luwero, Nakasongola, Nakaseke, and Mityana. These study settings have a high population density, urban-rural mix, cultural diversity, economic activity [16]. The prevalence of HIV stands at 6.2% compared to the country’s prevalence of 5.8% [17].

### Study population

The study population consisted of People Living with HIV (PLHIV) and receiving care under any of the models being evaluated, children and adolescents under the YAPS program, healthcare workers providing care to PLHIV under the DSDMs including community retail pharmacists, and the HIV focal persons.

### Sampling clients

Participants were purposely selected based on their involvement in HIV/AIDS service monitoring and supervision, service provision, leadership in DSDM or social support groups, and accessing ART through DSDM or social support groups. Clients were chosen from health facilities offering these models and social support groups, primarily government and private non-profit religious-based facilities. To be eligible for an interview, clients had to have received ART care for at least two years (FBIM clients) and one year (CCLAD, CDDP, and FTDR clients). The study included clients from two support groups: the YAPs model and the caregiver-led Dots model. Participants from diverse districts provided valuable insights into DSDMs for HIV care and treatment.

### Data collection

In-depth (IDI) and Key Informant Interviews (KIIs) were conducted in private settings within health facilities, community pharmacies, and district offices, lasting 30 to 45 minutes. Six experienced research assistants conducted these interviews guided by In-depth interview guides, FGD and KII guide topic tailored for each type of participant. Through these guides, the research moderators (research assistants) were able to obtain insight from various stakeholders regarding their experiences, perceived changes in knowledge, attitudes, and behaviors related to HIV prevention and treatment, as well as the program’s impact on the various groups receiving care under the different models, as well as their peers. Additionally, the research sought suggestions for improving the efficiency and sustainability of DSDMs and YAPS. These guides are attached as appendices I to VI. The focus group discussions (FGDs), key informant interviews (KIIs) with local leaders, and in-depth interviews (IDIs) with beneficiaries from various models, including YAPS, were conducted in Luganda, the local language used in the study districts. The research tools, initially designed in English, underwent expert translation into Luganda, followed by pretesting and validation during the training session preceding data collection. The data collection process for all participants was completed within a three-week period.

To obtain expert information about the DSDMs during the Key Informant Interviews (KIIs), we purposively selected stakeholders with expert knowledge of the DSDMs arrangement. To do this, we leveraged on the people with expertise such as health workers providing services, leaders within the differential service delivery models, HIV focal persons within the districts and counsellors. A total of 11 Key Informant Interviews (KIIs) were conducted across all districts. The KII respondents comprised: CCLAD leaders in Kassanda and Mubende districts (*n* = 2); CDDP focal persons in Mubende district (*n* = 1); Pharmacy operators involved in the Community Retail Pharmacy model in Kiboga, Mityana, and Nakasongola districts (*n* = 3); HIV/AIDS counselors in Kiboga, Mityana, Mubende and Nakasongola districts (*n* = 4); and the District HIV/AIDS focal person in Nakasongola district (*n* = 1).

Focus Group Discussions (FGDs) were conducted in six districts, with two FGDs comprising 8 participants each in Kassanda and Kyankwanzi districts, featuring People Living with HIV (PLHIV) receiving care under the CCLAD, CDDP, and FTDR, as well as YAPS and caregivers of children under directly observed treatment (DOTs). The focus group discussions facilitated a lively conversation, allowing participants to share experiences and perceptions and supplement their shared understanding of the DSDMs and the YAPS strategy [18]. Recruitment of the participants in the FGDs and the respondents was made possible through district HIV focal persons, who first explained the duration, purpose and the benefits of participating in the discussion groups to the participants before they were requested to partake in the FGD. Three FGDs with 8 participants each in Kiboga, Mityana, and Nakasongola districts, consisting of PLHIV receiving care under the Community Retail Pharmacy model. Two FGDs, one each in Mubende and Nakaseke districts, comprising YAPS participants. Another FGD was conducted in the Nakasongola district comprising 7 caregivers, providing care under the Care-Giver-Led DOTS intervention. By facilitating interactions and sharing experiences among PLHIV benefiting from the different DSDMs, the FGDs allowed in-depth insights into the cultural and service delivery contexts within the district and model contexts as well as enlist similarities and differences of perceptions and services deliveries within the different models. The FGDs ranged in duration from 90 minutes to 2 hours.

Relatedly, nine In-Depth Interviews (IDIs) were conducted with various stakeholders. Through the In-Depth Interviews (IDIs), we sought to obtain detailed insights into the experiences, perceptions, and outcomes of stakeholders engaged with the DSDMs in the study districts. The IDIs included: One IDI with a PLHIV in the CCLAD model in Mubende district (*n* = 1); Two IDIs with YAPS leaders in Kassanda and Nakaseke districts (*n* = 2); Two IDIs with youth PLHIV beneficiaries of the YAPS initiative in Kyankwanzi and Mubende districts (*n* = 2); Two IDIs with PLHIV under the CDDP model in Mityana and Mubende districts (*n* = 2); Two IDIs with PLHIV receiving ART services under the Community Retail Pharmacy model in Kiboga and Mityana districts (*n* = 2); Two IDIs were conducted with caregivers in Nakasongola and Nakaseke districts (*n* = 2) and Two IDIs with clients/participants involved in the Fast Track Drug Refill model in Kiboga and Kassanda districts (*n* = 2).

### Data analysis

All interviews, including individual KIIs, IDIs, and FGDs, were recorded in audio format, and transcribed in English by transcribers fluent in Luganda (the local language through which the interviews had been conducted). The KIIs involving healthcare workers, HIV-focused persons, and district managers were also audio-recorded and transcribed verbatim. The analytical process adopted an inductive thematic analysis approach [19]. The analysis commenced with the establishment of an initial codebook applicable to all interview types. The transcripts of the interviews and focus group discussions were imported into Excel. The two data analysists then read and reviewed the data so as to familiarized with it. Then, the coding process begun by assigning codes to data components that were later refined and reorganized into categories in another of the excel sheets. Codes were merged and refined as found necessary after which themes were identified by grouping related codes, analyzing their contents and assigning names and definitions to the themes. To ensure reliability, two researchers (SPK and RADDMO) conducted a trial run of this codebook by independently coding a random subset of transcripts. This step-by-step collaboration led to the refinement of their respective codebooks, bolstering consistency in coding. Every transcript underwent coding through a constant comparison methodology, with any disagreements about coding addressed through consensus. Themes progressively emerged, and codes were systematically aligned within these established themes. These themes were further categorized into 25 distinct codes, which were subsequently grouped into the five overarching themes mentioned above. The results are presented in line with the Consolidated Criteria for Reporting Qualitative Research (COREQ) [20].

## Results

The study comprised 63 participants, consisting of 30 males and 33 females. The participants included five health workers who provide HIV services, three HIV focal persons, and 55 HIV clients. The HIV client group consisted of 19 males and 36 females. The Focus Group Discussions (FGDs) included members from the CCLAD, CDDP, FBIM, and FDTR models, with each group consisting of 8–10 participants. Additionally, eight clients participated in client-led Directly Observed Therapy (DOT) and ten members were part of the Young Adolescent Peer Supporters (YAPs) program. The participants’ ages ranged from 13 to 55 years, with a mean age of 33.5 years. A detailed description of the respondents is presented in Table 1.

Data analysis revealed six primary themes: (i) Benefits of Differentiated Service Delivery Models (DSDMs), (ii) YAPs enhanced HIV care for adolescents Facilitating Disclosure, Counseling and group Support (iii) Challenges faced in implementing the models, (iv) Perceived barriers concerning the models, (v) Suggestions for improving DSDM services, and (vi) Issues related to DSDM sustainability.

## Benefits of differentiated service delivery models (DSDMs)

### Community-based DSDMs enhanced HIV care and treatment services

#### Community-based DSDMs foster high treatment adherence

DSDMs generally have positively impacted ART adherence and potentially improved viral load suppression. Feedback from key informants and clients stressed the effective enhancement of viral load monitoring through these DSDMs, ensuring timely assessments and subsequently reducing instances of unnoticed viral load increases.

*The DSDMs have streamlined viral load monitoring*,* addressing past issues where clients frequently missed scheduled appointments*,* causing result delays and elevated viral loads. As a result*,* these models offer benefits by ensuring the inclusion of all eligible clients for accurate viral load measurements* (KI, HIV Focal person).

In the CCLAD group, participant files rarely show red flags, indicating strong treatment compliance. Within this group, mutual support, open communication, and problem-solving contribute to high adherence, with participants actively seeking help from peers.

*In this model*,* group members diligently follow medication instructions*,* avoiding red marks. They collaboratively tackle adherence issues. They always remind each other to take their medication. Consistent communication and mutual support encourage shared knowledge and commitment* (IDI, CCLAD member).

However, among community models, health workers observe better adherence to drug pick-up appointments in the CDDP model, where services are more accessible to clients. Conversely, the CCLAD model experiences occasional missed appointments due to transportation issues.

*Among community models*,* I believe CDDP performs better because clients wait for us to administer the medications. This is unlike CLAD clients*,* who skip appointments due to transportation issues* (Health provider, IDI, Luwero district).

#### Community-based DSDMs enhance efficient ARV supply management

Healthcare professionals observe that guaranteed demand within community models has played a crucial role in enhancing the smooth and proficient distribution of medications across various community models. As a result, this improvement has positively impacted the management of ARV supply chains.

*Timely ordering has improved because we previously procured drugs whose consumption was low because we had to wait for clients to come to the facility. This resulted in lower consumption than now* (KII, HIV Focal person).

In connection with the above, stock redistribution has been practiced. Health facilities within the district redistribute medicines within health facilities to address shortages. Whenever a facility lacks supplies, it seeks out surplus medicines from others.

#### Reduction in travel time for community-based ART refills

CCLAD and CDDM have extended services to clients who previously had access difficulties. These include adolescents, disabled individuals, the elderly, and the economically disadvantaged. This has enhanced ART accessibility for vulnerable groups. Persons with disabilities, who initially faced problems accessing services, now report improvements, exemplifying a remarkable case in point;

*Persons with disabilities and the elderly encountered significant challenges in accessing HIV services. For example*,* three men from our village*,* two of whom are visually impaired and one with a leg injury*,* consistently required assistance to reach health facilities for ART. However*,* with the implementation of the CDDP model*,* they now efficiently receive services that were once inaccessible. This model is currently operating within their community*,* allowing drug distributors to even deliver medications to their homes* [FGD with CDDP clients, Mityana District].

#### Reduction in waiting time

The introduction of DSDMs has led to a substantial decrease in waiting times for PLHIV to access antiretroviral therapy. Notably, respondents utilizing the community retail pharmacy model reported an average waiting time of less than five minutes, while participants in the CCLAD model experienced minimal interactions with service providers. Furthermore, users of the CDDP model reported significantly reduced waiting times at drug distribution stations, enhancing the overall efficiency and accessibility of HIV treatment services.

*The implementation of DSDMs has additionally reduced patient waiting times. This change is due to the previous situation where patients would arrive and depart promptly*,* given the high volume that overwhelmed health workers’ capacity to attend to everyone* (KII, HIV Focal Person).

This is underscored in the view of a community retail pharmacy provider.

*The problem of extended waiting periods at the facility has been addressed. In the community retail pharmacy model*,* clients receive attention for a brief 5-minute duration* (KII, Community Retail Pharmacy Service Provider).

#### Enhanced flexibility in scheduling ART medication refills

The community-based DSDMs like CCLAD, CDDP, and retail pharmacy setups, created flexibility for ART medicine pick-ups by entrusting group members to pick drugs on their behalf. This innovative approach optimizes visit times, offering clients an effective and time-saving experience.

*Prior to the implementation of DSDMs*,* clients were required to reach the health center early in person and spend extended periods there*,* and depart in the late evening*,* approximately 6 p.m. The introduction of DSDMs has simplified the visitation timetable*,* rendering it more manageable and user-friendly for clients. Now*,* clients encounter a streamlined procedure*,* minimizing waiting durations and enabling a more effective clinic visit* (IDI, Client in Community Retail Pharmacy model).

Making medication collection more adaptable and minimizing appointment-related interruptions is crucial. Particularly, CDDP excels in this domain. It has significantly assisted clients in aligning their healthcare with their work and personal responsibilities. The CDDP beneficiaries maintain their employment while enhancing their socio-economic standing and overall well-being;

*In the CDDP*,* unlike health facility visits that consume a full day*,* community deliveries allow us to access services effortlessly. We sometimes have medicines delivered to our homes*,* saving transportation costs. We can complete household tasks or* tend to our gardens or manage our businesses *before heading to community drug distribution points. Even without transport*,* we save time and money*,* focusing on other responsibilities after shorter visits* (FGD, CDDP Client, Myanzi).

This strongly underscores the significance of flexible healthcare for effectively managing various life obligations. In alignment with this concern, another participant highlighted that;

*In the past*,* I had to give up gardening and hairstyling appointments to ensure an early arrival at the medical facility. But now*,* under the current system*,* I can prioritize my customers and conveniently visit the drug distribution point for medication collection and reviews* (IDI, CDDP member, Mityana district).

The CDDP operates with scheduled medicine delivery at a set time and location, likely leading to limited flexibility. Conversely, the CCLAD approach permits clients to rotate pickup times for better convenience. Qualitative insights confirm this, as interviews noted that DSDMs enable delegation of drug pickup to peers. This offers flexibility, allowing busy clients to delay pickup without issues, and ensuring accessibility when available.

*The CCLAD model has streamlined my planning. I can request a group member to collect drugs when my schedule is tight. Even if my turn is up*,* I can ask a teammate to do it for me while I do it the next round. Our cooperative culture in this model is great*,* allowing us time for our work. Previously*,* regardless of health or engagements*,* drug-pickup was mandatory*,* which isn’t the case now* (IDI, CCLAD Leader).

#### *Improved scheduling of appointments*

Service providers in DSDM models ensure clients are prompted to attend their scheduled appointments only when drugs are available.

*We have also made sure that we communicate with clients when ART medicines are available. This is so that they are readily picked and clients do not spend money when the drugs are not available* (KII, Community pharmacist).

This prevents needless travel due to the unavailability of drugs, which previously resulted in financial waste for clients;

DSDM models have demonstrated enhanced client attendance due to phone-based appointment reminders by providers. A community pharmacist confirmed this approach, stating that “*we call clients to ensure they attend ART appointments*,* resulting in improved appointment adherence*.” (KII, Community pharmacist).

#### *Social support in community models*

Within DSDMs, particularly in CCLAD, clients collaborate in groups to collect drugs and prevent missed pickups. During refill planning, if someone reports a high pill balance due to poor adherence, peers can offer support and address issues. In the CDDP model, with fewer clients per point, it’s easier to spot and follow up on missed pickups. This enhances accountability and support in the community.

*No one in our group is ever flagged red*,* signifying that they are struggling to improve their care. If there’s a challenge*,* we discuss it and find solutions. In a group*,* we support each other*,* reducing medication lapses. Being part of a group boosts motivation and encourages consistent medication intake. We educate and learn from one another*,* promoting adherence and sharing knowledge across different groups* (FGD with CCLAD, Kasambya).

Furthermore, CCLAD clients establish social protection support groups, pooling and saving financial assistance from their peers. They then utilize the savings to provide social support to members in need.

*As a group*,* whenever a member encounters difficulties*,* we visit and interact with them. While our initial intention was to contribute 5*,*000 Ugandan shillings each (equivalent to 1.5 US Dollars)*,* we later revised this amount to 1*,*000 shillings (about 0.3 US Dollars) due to financial constraints among members. However*,* during our gatherings*,* we raise these funds*,* which we then allocate to members in need to help them buy essentials like milk for better nutrition. This gesture serves to boost the needy members’ morale and motivate them to prioritize their nutritional needs and seek necessary medical care* (FGD with CCLAD, Kasambya).

#### *Reduced stigma*

The participants reported that DSDMs have been effective in reducing stigma to a significant extent, by providing discreet access to medication and promoting a more confidential and patient-centered approach to healthcare delivery. Unlike clinics, communal setups ensure private medication pick-up. The implementation of community-based models has yielded a significant increase in PLHIV engagement, facilitated support for disclosure, stigma management, and treatment acceptance. The confidentiality inherent in the DSDMs has been instrumental in reducing stigma, fostering a safe and trustworthy environment for individuals to access essential health services.

*ART recipients encountered community isolation and stigma*,* were clinic visits automatically implied HIV status. The innovative DSDM approach curbs stigma*,* simplifies disclosure*,* and offers treatment*,* counseling*,* and understanding* (KII, pharmacy service provider).

#### *Reduction in congestion at the health facility*

Previously, health facilities were always crowded during drug pick-ups and patient reviews. This impacted patient well-being and increased the risk of contracting contagious diseases. The findings highlight the role of DSDMs in alleviating overcrowding in health facilities during the process of refilling ART medications. Scheduled clinic appointments curbed congestion by assigning specific times, reducing wait times, and enhancing resource distribution.

*Most clients were shifted to community models*,* leaving few in facilities. The CDDP and CCLAD models outperform FTDR and FBIM in curbing queues and infections. However*,* FTDR facility clients*,* often on multi-month appointments*,* encounter less congestion compared to FBIM due to widespread appointment scheduling* (KII, HIV focal person).

*The introduction of DSDMs has effectively reduced overcrowding. This has reduced TB and COVID-19 transmission risks. Previously*,* patients with different ailments*,* including coughing and fever*,* encountered delays and suboptimal prioritization. The introduction of DSDMs has successfully resolved these concerns by forming groups and streamlining processes*,* ultimately benefiting the community* (CCLAD Leader, Kasambya).

#### *Enhancing communication with non-treatment-adherent clients*

With the DSDMs, healthcare providers can now easily connect with clients, whether facility-based or community-based, who may have previously missed appointments. This represents a significant change from the past, resulting in enhanced adherence to treatment protocols.

*Transport challenges impede FBIM clients*,* including pregnant women and children under grandparents’ care*,* from consistently attending appointments. Health workers are*,* however*,* assisted with transport when visiting community-based DSDM patients. This motivates health workers to engage with non-adherent clients* (KI, HIV Focal person).

## Benefits realised with facility-based DSDMs

### Adaptable care provision within the facility-based models

Healthcare providers in the facility-based model (FBIM) have implemented novel approaches, such as extending service hours and peer medication delivery, to enhance service delivery. This aims to accommodate clients who avoid crowded health facilities or have work commitments during regular hours.

*Delivering services at a facility can present challenges*,* as it necessitates clients physically visiting the facility. However*,* we have implemented solutions to this. To accommodate clients who are unwilling to attend during regular working hours*,* we offer extended appointments during less congested times. The distribution of drugs is further facilitated by peers. We maintain thorough client records that are consistently reviewed and revised. Details such as the locations of stable clients are included in these records*,* allowing appointments to be scheduled efficiently* (KII, HIV Focal person).

As a result, this innovation in drug collection through FBIM’s adaptable approach improves clients’ work-life balance, allowing PLHIV to effectively manage their commitments and allocate time accordingly.

The option to delegate medication pickup through DSDMs grants flexibility and eases busy schedules. This is especially beneficial for those with demanding routines or limited mobility, ensuring timely access. It’s recommended to enhance DSDMs through clear communication, tracking, and feedback. Community pharmacies offer daily accessibility, letting clients pick up medicines when convenient. This saves time and aligns with their schedules.

*My crops would be damaged by monkeys and birds because I spent hours at the facility as a farmer. But now*,* I wait until the evening*,* around 6 p.m.*,* to collect my medication before 8 p.m.*,* as instructed* (IDI, Community Pharmacy, Mityana).

### *Reduced exposure to infectious disease*

Within the DSDMs arrangement, HIV clinics have introduced an appointment scheduling system that designates specific days for clients from various models. This not only streamlines appointments but also minimizes infection exposure, as clients of distinct models often exhibit differing HIV statuses in terms of viral load suppression and opportunistic infections.

*ART services are scheduled on distinct clinic days*,* considering age*,* health conditions*,* and models. Mixing of clients is avoided to ensure appointment confidence and ample time is allotted to encourage adherence. For example*,* in our district*,* FTDR and FBIM clients visit on Monday and Thursday*,* breastfeeding mothers*,* teenagers*,* and children on Tuesday*,* while non-suppressors*,* ART initiators*,* and TB patients attend on Wednesday* (KII, HIV Focal person).

### *Improved hospitality of health workers*

The DSDM model excels at delivering services with notable hospitality, as evident from client feedback indicating extra attention and enhanced appointment adherence. The community pharmacy model, in particular, is renowned for its caring approach, with medical staff providing clear instructions and attentive care. As per insights shared by an interviewed community pharmacist, “*pharmacists emphasize that showing love and care to HIV-positive individuals is vital for improved cooperation*,* appointment attendance*,* and ART treatment adherence*”.

## YAPs enhanced HIV care for adolescents facilitating Disclosure, Counseling and group support

### *YAPS mentors facilitate HIV disclosure among adolescents*

YAP counselors assist adolescents who are hesitant to share their HIV status with others to do so. After the disclosure, the adolescents will offer reminders to colleagues on treatment, help each other alleviate stress, improve understanding of treatment, and increase engagement with healthcare providers.

*Most adolescents find HIV status disclosure daunting. In my role as a YAP mentor*,* I extended support to those seeking to share their status with friends. I assessed their confidence levels to counter stigma. After disclosure*,* some have testified that disclosure has enhanced medication adherence*,* describing it as noticeably easier* (IDI, YAPS member).

Disclosure of their HIV status enhances medication adherence among many adolescents, as it offers emotional support, diminishes stigma, and contributes to better medication adherence.

*In the past*,* misconceptions about drug use and a fatalistic belief in the inevitability of death if one is HIV positive hindered many adolescents from adhering to ART guidelines. However*,* with our peer counselling under the YAPs*,* attitudes have evolved*,* and a positive shift has occurred*,* leading to increased adherence to appropriate drug use protocols.* (IDI, YAPS member).

A respondent revealed that while negative social experiences in play settings often induce fear and distress during childhood and adolescence, involvement in YAPs and counseling equips young individuals with HIV with resilience and coping skills to effectively handle such situations.

*Previously*,* I felt fearful on the playground due to others controlling my interactions and preventing me from being with friends. Now*,* I remain quiet instead of reacting emotionally to hurtful remarks as I did before* (IDI, adolescent under YAPS).

## Challenges associated with DSDMs strategy

There are several challenges encountered in the implementation of the DSDMs and the YAPS strategy. These are presented as.

### Challenges associated with community-based DSDMs (CCLADD and CDDP)

#### *HIV status disclosure may not have improved*

The findings highlight a lack of significant progress in HIV status disclosure, especially among those in the community retail pharmacy and CCLAD models. In a deep interview, a CCLAD leader voiced concern about members hiding medications in fellow members’ homes due to undisclosed HIV status to partners. “*Some members keep their medications in others’ homes and take them from there*.” In the community pharmacy model, a community pharmacy service provider linked non-disclosure to pharmacists not addressing disclosure worries and engaging with clients on this crucial topic. She said, “*We only provide them with ART medications*,* so we have not delved into whether they feel comfortable sharing their HIV status with friends and family.”* (Key Informant Interview).

Due to concerns about revealing their HIV status, some eligible individuals decline participation in various community service delivery models. They opt for facility-based approaches, fearing that joining a community group could lead to inadvertent disclosure of their status to others in the community.

*Upon evaluating the enrollment of clients in the community model*,* it’s evident that enrollment is limited due to many individuals hesitating to disclose their HIV status. In the case of the CCLAD model*,* individuals must inform their assigned group members about their involvement*,* yet many are still hesitant to comply* (KII, HIV focal person).

#### *Delayed identification of opportunistic infections*

Clients in certain models, like CCLAD and community retail pharmacies, disregard timely medical consultation for symptoms and agitate for free treatment at community pharmacies. This hinders timely monitoring and opportunistic infection identification, potentially increasing opportunistic infection rates and compromising their well-being.

*Within the community retail pharmacy model*,* specific individuals acquire opportunistic infections*,* yet lack the financial means for treatment. They resist health facility referrals*,* instead pressuring community pharmacists for free medications via the DSDM arrangement*,* which contradicts established agreements. In a particular instance*,* an individual became ill without seeking medical help or informing the facility or clinician. As a result*,* a tuberculosis diagnosis was delayed*,* and he eventually died just after he was taken to the hospital* (KII, Community Pharmacy Service Provider).

#### *Non-adherence to tuberculosis treatment*

Tuberculosis (TB) treatment regimens completion is a challenge for some PLHIV enrolled in the community-based models. Among the primary obstacles are high mobility, which makes adhering to medication schedules and accessing care difficult, and logistical obstacles to accessing medications, which can result in interrupted treatment or failure of the treatment due to their transient nature.

*The completion rate is poor for TB*,* and this is due to client mobility. The majority of our clients are charcoal makers and fishermen who are always on the move and fail to attend appointments and treatment*. (KII, HIV Focal person).

### *Some PLHIV in the community models occasionally skip their scheduled appointments*

During rainy farming seasons, some clients face difficulties meeting review appointments and adhering to their medication when they relocate to other places of work.

*In the farming season*,* individuals relocate to Kyegegwa*,* Kyenjojo*,* and Fort Portal*,* resulting in appointment gaps. As they engage in seasonal work*,* treatment adherence is affected by limited medication supplies*,* yet there is no replenishment. Although challenging*,* the organization persists in providing long-term medications despite clients being away for months* (KI, HIV, focal person).

#### *Fears related to lack of confidentiality in community DSDMs*

Some PLHIV receiving ART under facility-based models although eligible for transition to community-based service delivery models have declined to do so. However, there are breaches of confidentiality within the CCLAD model, whereby some PLHIV have at some occasions shared sensitive information about their peers and group activities to others outside of the model. This has affected trust and hindered potential enrollees from joining the model, fearing unauthorized disclosure of their personal health information. An HIV focal person shared his insights on this matter, stating;

*When you look at our enrollment at the community level*,* you will notice that it’s somewhat low since many people do not want to disclose their HIV status. If you participate in the CCLAD model*,* you must tell your friends with whom you will be in the group. However*,* many people do not want to do so* (KII, HIV Focal person).

Furthermore, the CDDP model also poses confidentiality risks, as PLHIV’s visits to drug distribution points can be observed by community members, potentially fueling rumors and inadvertently some members disclosing their HIV status, thereby compromising their right to privacy and confidentiality. A female member of CDDP model has this to say;

*The CDDP model presents a challenge to maintaining confidentiality in community-based care. The PLHIV in this model may be observed by community members visiting health facilities on scheduled appointment days and collecting medications. This may potentially fuel rumors and inadvertently disclose their HIV-positive status thus compromising the privacy and confidentiality.* (KII, Health worker).

The aforesaid challenges have resulted in a preference among potential enrollees to opt for continued ART care within facility-based models, rather than transitioning to community-based models. This decision has consequently led to sustained congestion in some health facilities, as a significant proportion of PLHIV seek to maintain their care within these settings, thereby straining facility resources and capacity.

#### *Poor categorization and Placement of PLHIV into Community DSDMs*

Poor filing and record keeping on the part of the medical staff occasionally results in poor patient classification. Due to this, PLHIV are placed in groups to which they do not belong. As an illustration, some PLHIV are placed in the category of “stable clients” when they are not stable. In contrast, others who are stable are erroneously placed in the “unstable clients” category. This jeopardizes their care.

*Poor client categorization posed a problem because it resulted in challenges of enrolling stable clients into the community model. Client files are supposed to be marked with a yellow or red tag. However*,* occasionally files are not marked*,* making it impossible to identify stable or unstable clients* (KI, HIV focal person).

### Challenges faced by health workers offering ART services under the DSDMs model

#### *CPLHIV lack of commitment to pick drugs from the pharmacies*

One of the challenges faced by health workers in the community retail pharmacy model is encouraging patients to adhere to their treatment regimens and keep their appointments. Despite regular reminders from community pharmacists, some patients demonstrate a lack of commitment to their treatment, posing a significant obstacle to successful health outcomes and highlighting the need for additional support strategies to enhance patient engagement and motivation.

*We have a difficult time always reminding patients because if we do not*,* they will forget about their appointments and not show up at the scheduled time to pick up their ART medications*. (KII, Community pharmacist).

#### *PLHIV under the Pharmacy Model expect free non-HIV care*

Health workers in the community retail pharmacy model face a challenge when PLHIV under this model with comorbidities expect free treatment for non-HIV related illnesses. When the pharmacists request payment for these services, this leads to conflicts. The PLHIV often argue that this care should be covered under the community-led pharmacy model contrary to the memorandum of understanding stipulations. This highlights the need for clear communication, boundary setting, and reimbursement protocols to avoid misunderstandings and ensure sustainable service delivery.

*Some PLHIV come with other diseases and want to get treatment for them at no cost*,* thinking that we give all treatment for free*,* yet it’s only ART services*,* so when you ask for money in order to treat those other diseases*,* they say they don’t have money and request to get treatment another time*,* whereby they don’t even come*,* which I think is leading to poor adherence* (KII, Community pharmacist).

#### *PLHIV providing wrong phone numbers*

The community retail pharmacists encounter significant challenges in the form of inaccurate, erroneous, or unreliable contact information provided by PLHIV enrolled in this model. The inadequacy of reliable phone numbers hinders the ability to conduct effective follow-up communications, track medication adherence, and monitor patient progress, thereby impeding the provision of comprehensive patient support and retention in care.

*We face the challenge of clients giving us wrong information*,* such as wrong phone numbers. Although we have a tracker*,* we really find it challenging to follow up with such clients and help them* (KI, HIV focal person).

#### *Inaccurate data*

The accuracy of client information across various models has been compromised due to the incomplete data in the electronic medical records (EMR) system, which lacks essential patient information. Consequently, the reliability of the data in the models is questionable, and the maintenance of accurate and comprehensive records has become a significant challenge. A health worker noted that; “*There was an issue with the data*,* especially the HCT data*,* which occasionally wasn’t entered into the EMR system”*. This has hindered the effective management and coordination of care.

#### *Lack of transport*

Whereas healthcare providers and HIV-focal persons aim to occasionally conduct supervisory visits to monitor the progress of PLHIV under their care, more so those enrolled in the community-based models, they frequently encounter challenges in accessing reliable transportation. This hinders their impedes conduction of the routine follow-up visits in order to track and provide adequate support to these persons. This, therefore, compromises the treatment outcomes of some of the PLHIV especially those who may not be adhering appropriately to the treatment.

### Challenges experienced by the YAPS members

#### *Discontinued support services*

When the YAPS programme had just been introduced, YAPS members would be provided with transport and food provisions on ART days. However, the discontinuation of this support resulted in a significant YAPS members encountering difficulties in accessing healthcare facilities for essential care. Those who manage to come for the ART services after being rallied by their peers to do so get demoralized due to hunger resulting from no food given after long waiting times. Consequently, some YAPS are missing their treatment, failing to adhere thus leading to failure to maintain optimal health outcomes.

*Many youths mobilized under the YAPS initiative come from home without food. The youth need preparations for ART Day*,* such as food. We used to do that. I am not sure why it was dropped. They used to transport them and give them food.*

#### *ART day challenges*

In the view of some YAPS, the current ART Day processes lack effective streamlining, which results in prolonged waiting periods and a suboptimal experience for pediatric patients. Significant delays in retrieving files result in longer wait times for young patients. Delays are resulting in physical and emotional distress for the children, manifesting as hunger and upset emotions, with some even becoming so distraught that they cry.

#### *Complex family dynamics*

Certain children are residing in households with intricate family structures, which are hindering their ability to receive optimal Antiretroviral Therapy (ART) care. Specifically, those living with non-biological caregivers or guardians may be at risk due to the caregivers’ lack of awareness regarding the children’s HIV status. Additionally, in some instances, biological parents may conceal their HIV status from non-biological co-parents or guardians. These non-biological parents or guardians may not be equipped to provide adequate care, especially during medical appointments. This creates a barrier to effective care and support for the children. This complex family dynamics can lead to inadequate medication management, reduced adherence, and compromised health outcomes for the affected children.

### *Concealment of HIV i*dentity *by some adolescents*

Due to fear of judgment or rejection, some adolescents living with HIV hide their positive status and whereabouts from colleagues and friends in the community. This creates a challenge to offer them support when it would be needed.

*Some teenagers living with HIV may keep their status and whereabouts secret*,* and may even discontinue their antiretroviral therapy (ART) after leaving home. Furthermore*,* they may not disclose their status to their colleagues.*

In other cases, children staying with parents where one of them may not be their biological parent may have their HIV status concealed from the non-biological parent. This makes it challenging to provide adequate care for the child, especially when appointments are scheduled while the child is in the care of the non-biological parent who is unaware of the child’s status.

### *Negative coping mechanisms*

Although the YAPS initiative has pioneered important improvements in mitigating stigma among adolescents and children, some children and adolescents still struggling to cope with stigma have adopted rather unhealth coping strategies such as silence and withdrawal to avoid emotional distress and conflict. This results into reduced access to social support and connection eventually affecting their wellbeing.

## DSDM’s sustainability concerns

### *Funding threatens sustainability of the community DSDMs*

The implementation of DSDM models is primarily funded by external agencies through local or international implementing organizations. If current funding stops without viable alternatives, the ongoing sustainability of community DSDM initiatives could be jeopardized.

*“Community-oriented HIV service delivery*,* such as CDDP*,* CCLAD*,* and community pharmacies*,* necessitates financial support. Incentives are essential for health workers*,* and transportation assistance is crucial for effective service provision. Nevertheless*,* funding to ensure the continuation of these endeavors remains unclear”* (KII, HIV focal person).

### *Government Health personnel’s insufficient interest in supporting DSDMs*

The provision of DSDM models and HIV care in health facilities primarily operates through a vertical program structure funded by donors via implementing partners. In the examined districts, Mildmay Uganda served as the implementing partner at the time of the study, seconding staff to these facilities and supporting allowances for government health workers involved in outreach services more so for supervision of community based DSDMs. However, doubts arise about the models’ sustainability if they are exclusively offered by government health workers without the monetary incentives. The concern stems from government health workers displaying reduced enthusiasm for uncompensated facility-based services, which could potentially worsen if incentives for outreach services are removed in the future.

*Public health workers hold negative views about facility-based DSDM ART services. They prefer community-based models*,* where they are paid some allowances which may not be the case in future with reduced funding* (KI, HIV Focal person).

### *Limited YAPS mentors guiding adolescents*

YAPS mentors have expressed concerns about their limited numbers. This impacts the quality of their services when they need to be absent from their communities for extended periods of time. Additionally, they are worried about the insufficient recruitment and training of new YAPS mentors who could fill their roles as they approach the program’s age limit.

*Maintaining a continuous YAPS counselor presence in the community is crucial. Instances occur where clients get used to a YAPS counselor*,* but once such a counselor is away for any reason*,* their work is missed. The lack of trained YAPS mentors as we near 24 years of age and transition into adulthood without being replaced is also concerning* (IDI, YAPS Mentor).

### *Poor adolescents and children’s ART-day preparations*

Most review days are inadequately planned, resulting in delayed file access and hunger. Several adolescents and children attend hungry, enduring the whole day without meals, causing distress. They propose offering snacks during waiting times. Additionally, some struggle with long distances to medical centers, occasionally missing appointments due to transport issues. To address this, providing transportation assistance is suggested to minimize appointment no-shows.

*Several adolescents and children come to ART days without having had a meal at home. High attendance*,* coupled with file retrieval delays*,* resulted in long waiting times*,* leading to severe hunger and frustration. Some children even cry due to this issue. Previously*,* adolescents used transportation reimbursements to purchase food*,* but the reason for discontinuing this practice is uncertain* (IDI, YAPs mentor).

In order to ensure the well-being of the youth during ART Day, it is crucial to enhance preparations, including food provision and logistical arrangements as stated by a YAP counsellor that “*What can help is empowering us come for our clinic appointments is to give us food at the health facility on clinic days*.”

### Suggestions for improving the DSDM approach

#### *Introduce social protection measures within all the community DSDM groups*

Participants suggested that, apart from enhancing healthcare access, community-based DSDMs should focus on creating social protection measures for sustainability beyond donor funding. If donor funding is withdrawn, social support in the form of community-based health insurance schemes could fill funding gaps by involving group members.

*At the outset*,* our main objective was to form groups to improve ART treatment access and overall well-being. Yet we gradually observed the significance of addressing wider issues*,* including social and economic empowerment. We recognize the need to assist individuals beyond medication*,* covering transport and communication as well. This is intended to empower community retail pharmacists to continue functioning if donor funding for the DSDMs is withdrawn* (KI, community retail pharmacist, Mityana District).

#### *Injectable ART for all PLHIV in all DSDMs*

HIV clients prefer injectable ART due to its possibility of improving adherence during travel or in situations where oral medication might be forgotten. Injectable ART also addresses medication errors such as underdosing or overdosing, as illustrated in the IDI with a client in the CDDP model that;

*The availability of injectable ART could alleviate anxiety related to missed oral doses. Injectable ART is likely to prevent risks like forgetting oral doses during unexpected trips*,* ensuring uninterrupted journeys* (IDI, CDDP member, Kabuwambo).

### *Parental monitoring of adherence to enhance YAPS activities*

Healthcare professionals recommend avoiding unsupervised vacations for HIV-infected children to improve medication adherence. Parents or caregivers must accompany children on ART while they travel to ensure proper medication administration as commented by a YAP mentor that; “*It is not advisable for young children who cannot self-administer medication to go on vacation outside of their parents’ or caregivers’ supervision. Parental supervision is recommended for children*.” (IDI, YAPS mentor). The idea behind this is to ensure that those familiar with the children’s care continue to take charge of medication administration so that deviations from prescribed instructions do not affect adherence, as may happen if this role is delegated to others.

## Discussion

Our study’s findings corroborate the notion that the existing pharmaceutical supply chain framework is not adequately equipped to support effective DSDM implementation [11]. The persistent drug shortages in low- and middle-income countries pose a significant hurdle to the successful implementation of Differential Service Delivery Models (DSDMs), underscoring the urgent need for innovative medication distribution strategies [21]. To address this challenge, exploring diverse stock redistribution approaches, including cutting-edge technology-based solutions such as real-time tracking and predictive analytics, is crucial. Moreover, fostering seamless communication among healthcare facilities, storekeepers, and supply chain managers can facilitate efficient stock redistribution. Promisingly, healthcare managers have taken proactive steps to address this issue by redistributing medicines among healthcare facilities, thereby enhancing availability and accessibility in the studied areas. This strategy aligns with Uganda’s broader goal of establishing a robust and dependable supply chain management system, mitigating drug scarcities, and ensuring the efficient and effective supply of medications [4].

The successful implementation of HIV programs hinges on healthcare providers displaying a positive and respectful attitude towards People Living with HIV (PLHIV), a crucial factor emphasized in previous studies [6]. Our respondents echoed this sentiment, praising healthcare providers for their approachable and hospitable nature. The feedback on hospitality and attention in Differential Service Delivery Models (DSDMs) highlights the vast potential for expanded implementation, corroborating findings from previous research [22]. By prioritizing client-centered hospitality, healthcare providers can cultivate trust, diminish stigma, and enhance treatment adherence, ultimately contributing to successful treatment outcomes and HIV prevention [23]. Strategies such as ensuring medication availability, timely communication, and welcoming health workers have proven effective in reducing unnecessary travel, boosting adherence, and improving client satisfaction, thereby preventing missed visits and ensuring treatment continuity.

This study’s findings reveal a significant dichotomy in waiting times, with community-based models (FBIM, CCLAD, and CDDP) and facility-based FTDR demonstrating reduced waiting times, while facility-based FBIM continues to struggle with prolonged delays. The success of community-based approaches and FTDR in decreasing waiting times underscores their effectiveness in enhancing service quality [24]. Innovative strategies, such as revised visiting schedules, have also significantly reduced waiting times, improving clients’ time management and reducing congestion, infection risk, and discomfort. Investing in these models can further improve access and streamline service delivery, while addressing the persistent limitations in FBIM is crucial to advancing HIV care [25].

This study demonstrates the profound impact of Differential Service Delivery Models (DSDMs) on enhancing Antiretroviral Therapy (ART) adherence and viral load suppression among individuals living with HIV. By addressing issues such as missed appointments and delays, and streamlining viral load monitoring, DSDMs have proven to be a game-changer in HIV care. Notably, the C-CLAD model has excelled in promoting treatment compliance, thanks to the power of mutual support and open communication within its community, corroborating previous research on the efficacy of community-based models like C-CLAD [26]. Although transportation challenges occasionally hinder C-CLAD’s performance, the CDDP model stands out for its accessible treatment options, potentially boosting adherence rates [27]. Our findings underscore the critical importance of tailored DSDMs in optimizing ART adherence and viral load management. Moreover, our study reveals that community-based DSDMs play a vital role in reducing HIV-related stigma by providing discreet services, promoting privacy, fostering support networks, normalizing care, raising awareness, engaging communities, and empowering individuals [28]. By doing so, DSDMs contribute significantly to creating a more inclusive and stigma-free environment for those living with HIV.

Our findings reveal a disconcerting lack of progress in HIV status disclosure among community retail pharmacies and CCLAD clients, contradicting Kintu et al.‘s (2021) observations of increased openness among community model participants [12]. However, our results align with earlier research highlighting the persistent challenges in promoting disclosure [23]. The inability to enhance HIV disclosure among clients underscores significant barriers, with far-reaching consequences for transmission prevention, treatment access, stigma reduction, and public health efforts [26]. Moreover, non-disclosure has driven clients back to facility-based models, exacerbating congestion and related issues. To address these challenges and achieve effective HIV prevention and care strategies, fostering candid discussions about HIV status among clients is key [29]. To facilitate this, we recommend strategies such as enhancing privacy, education, peer support, and gradual exposure, as well as voluntary disclosure. Additionally, community pharmacist training, improved counseling, informative resources, and pharmacist-support group collaboration can help reduce fears, encourage participation, and improve care access for individuals with HIV.

Concerns have been raised about the potential delay in detecting opportunistic infections through community-based models. Consistent with previous research, our findings suggest that prolonged gaps in interactions with healthcare workers within community differential delivery models may hinder access to healthcare for other illnesses, notably Tuberculosis (TB) [11, 29]. To optimize the CCLAD model’s effectiveness in preventing opportunistic infections, healthcare providers must educate clients on seeking prompt medical attention for even minor health changes or ailments. Our results also highlight the need for implementing opportunistic infection screening and health education sessions for clients in this module. Furthermore, the resistance of clients in the retail pharmacy model to referrals for other infections underscores the importance of aligning client expectations with community retail pharmacies’ roles in infection treatment. To address this, training programs for clients and pharmacists can enhance awareness about appropriate care-seeking and refine the referral process for suspected infections, ultimately improving health outcomes.

Various challenges impacting effective service delivery under the DSDM have been identified, encompassing issues such as appointment keeping and medication adherence during agricultural seasons, unmonitored breaks disrupting medication routines, and hesitance among government health workers to continue providing DSDM-based ART services. Addressing these concerns requires educating clients about consistent adherence and providing extended medication supplies. Creative refill methods and follow-up visits or calls are essential to managing adherence challenges. In the case of young HIV-infected children, challenges with self-administration during the holidays highlight the need for careful planning with healthcare providers. This involves identifying caregivers, developing travel medication strategies, and maintaining communication. Encouraging a positive attitude among health providers, addressing incentives, and advocating for funding can bolster health workers’ commitment to delivering care under various DSDMs.

This study highlights the imperative need to ensure the sustainability of community-based Differential Service Delivery Models (DSDMs) by exploring strategies to mitigate the impact of potential donor funding reductions. The reliance on donor funding for DSDMs in sub-Saharan Africa, including Uganda, raises concerns about long-term viability [30]. To address this, we recommend prioritizing self-sustaining measures, such as community-based health insurance models, which align with previous research [12]. Empowering group members to take ownership of these initiatives can help counteract the effects of funding cuts [9]. A pilot study is needed to determine the impact of client-financed ART delivery on acceptance and treatment outcomes in the CDDP model, which could potentially become self-sustaining by adopting social solidarity financing approaches.

The findings indicated that clients show a preference for injectable long-acting ART, citing reasons like avoiding concerns about pill visibility to others and alleviating worries about daily oral treatment adherence. Injectable ART offers convenience, stigma reduction, and improved adherence, addressing daily challenges [31]. Injectable ART can decrease the number of health facility visits needed, reduce congestion, streamline services, and maximize healthcare resources. This makes healthcare more accessible to most patients and leads to better experiences and outcomes [32]. This has the potential of improving service deliveries under the DSDMs especially for clients shunning models that may lead others to know their status [33]. It also may lead to reduction of congestion at the health facilities Integrating injectable ART empowers healthcare providers and policymakers for enhanced adherence, outcomes, and satisfaction, aligning with UNAIDS’ goals for comprehensive HIV care improvement [34]. However, to achieve this, it may require removing bottlenecks that hinder its access and monitoring its implementation.

This study highlighted the significant role of Young Adolescent Peer Supporters (YAPs) in helping hesitant young individuals disclose their HIV status. Status disclosure is crucial for effective HIV management, and YAPs can contribute to reducing stigma and improving medication adherence. The involvement of YAPs proves pivotal in enhancing the well-being of young HIV-positive individuals, emphasizing the need for comprehensive HIV programs targeting this demographic. Integrating counseling and support services for HIV disclosure with YAP mentors can be effective. Youth-led initiatives and support groups can amplify medication adherence and health outcomes. The YAPS program has influenced adolescents to adhere to correct ART guidelines, dispelling misconceptions and encouraging proper medication use. Addressing current challenges faced by YAPS mentors, like mentor shortages, necessitates strategies such as improved recruitment, mentorship approaches, and community involvement to ensure a seamless transition as mentors age out of the program.

### Conclusion

Our study’s findings illuminate the complex challenges and opportunities influencing the effective implementation of Differential Service Delivery Models (DSDMs). Community-based models and facility-based Fast-Track Drug Refills (FTDR) demonstrate reduced waiting times, improved treatment adherence, diminished stigma, and enhanced overall health outcomes. However, persistent challenges in Facility Based Individual Management (FBIM) model, including lengthy waiting times, hesitant HIV status disclosure, and poor health worker attitudes, hinder DSDM success. To reform HIV care and improve the lives of those affected, tailored, patient-centered strategies within DSDMs are crucial. Moreover, integrating Young Adolescent Peer Supporters (YAPs) and addressing their needs will optimize outcomes for adolescents, while injectable ART preferences offer opportunities to alleviate health facility congestion and boost adherence.

### Recommendation

Introducing cross-subsidization and strengthening social support within community-based DSDMs is crucial. To enhance HIV client adherence, community retail pharmacy models must improve communication, visitation schedules, and pharmacist training. Training group leaders and addressing negative perceptions among public health employees is necessary. Increasing YAPs mentors and enhancing recruitment strategies is vital. Preparations for ART days, continuous awareness campaigns, and specialized counseling are indispensable. Building capacity in rural pharmacies to offer discreet services will accommodate clients’ preferences. A pilot study on client-financed ART delivery to community pick-up points within CDDP is recommended to assess model acceptance, cost, and ART outcomes.

### Study limitations

When interpreting the study results, it is important to consider the following limitations: The community pharmacy model was active in only three of the eight districts at the time of the study. These districts were primarily in urban areas and had a limited number of facilities. Additionally, PLHIV across the various models had different exposure durations to the services. Therefore, their opinions are presented based on the time they spent in each model, potentially leading to an overrepresentation or underrepresentation of perceptions. In addition, this subjectivity may lead to others interpret findings differently. Given that this is a ‘qualitative phenomenological research design’, the findings cannot be generalized beyond this context. They are also limited to the perceptions and experiences of respondents and their descriptions. Besides, it is not easy to determine the cause-and-effect relationship with this approach.

### Electronic supplementary material

Below is the link to the electronic supplementary material.

Supplementary Material 1

Supplementary Material 2

Supplementary Material 3

Supplementary Material 4

Supplementary Material 5

## Data Availability

All the data and material for this study has been presented in the article.

## Abbreviations

ART: Antiretroviral therapy
CDDP: Community Drug Distribution Point
CCLAD: Community-Client-Led Drug Distribution
DSDMs: Differential Service Delivery Models
FBIM: Facility-Based Individual Management
FTDR: Fast-Track Drug Refills
YAPS: Young Adolescent Peer Supporters

## References

[1] Fatti G, Ngorima-mabhena N, Tiam A, Tukei BB, Kasu T, Muzenda T, et al. Community-based differentiated service delivery models incorporating multi-month dispensing of antiretroviral treatment for newly stable people living with HIV receiving single annual clinical visits: a pooled analysis of two cluster-randomized trials in. J Int AIDS Soc. 2021;24(S6):24–9. 10.1002/jia2.25819 PMC8554219 34713614

[2] Miyingo C, Mpayenda T, Nyole R, Ayinembabazi J, Ssepuuya M, Ssebuwufu EM et al. HIV Treatment and Care of Adolescents: Perspectives of Adolescents on Community-Based Models in Northern Uganda. HIV/AIDS - Research and Palliative Care. 2023;15(March):105–14. 10.2147/HIV.S405393 PMC10015975 36938317

[3] Komujuni H, Tumwikirize J. Guide to Onsite Preparation for differentiated HIV Care and Treatment services using the Community Client Led ART Delivery Model: experience from Seven Public Health Facilities in Uganda. University Research Co., LLC (URC): Vol. USAID ASSI; 2017.

[4] The Republic of Uganda. Implementation guide for Differentiated Service Delivery models of HIV services in Uganda. Volume 20. Kampala: Ministry of Health; 2020.

[5] Mujjuzi I, Mutegeki P, Nabuwufu S, Wosukira A, Namata F, Alayo P et al. Care Burden and Coping Strategies among Caregivers of Paediatric HIV / AIDS in Northern Uganda: A Cross-Sectional Mixed-Method Study. AIDS Res Treat. 2021; 2021:1–14. 10.1155/2021/6660337 PMC8452441 34552767

[6] Oryokot B, Kazibwe A, Kagimu D, Oluka AI, Kato D, Miya Y, et al. Improving retention and HIV viral load suppression among adolescents living with HIV in TASO Soroti and TASO Mbale centers of excellence using operation Triple Zero model: a before and after study protocol. Implement Sci Commun. 2023;4(1):1–12. 37308985 10.1186/s43058-023-00449-9 PMC10259809

[7] PEPFAR UGANDA. Uganda Country operational plan (COP) 2021 Strategic Direction Summary. Kampala: Pepfar; 2021.

[8] Lorenz R, Grant E, Muyindike W, Maling S, Card C, Henry C, et al. Caregivers’ attitudes towards HIV Testing and Disclosure of HIV Status to At-Risk children in Rural Uganda. PLoS ONE. 2016;11(2):e0148950. 26881773 10.1371/journal.pone.0148950 PMC4755560

[9] Tourneau N, Le, German A, Thompson R, Ford N, Shwartz S, Beres L, et al. Evaluation of HIV treatment outcomes with reduced frequency of clinical encounters and antiretroviral treatment refills: a systematic review and meta-analysis. PLoS Med. 2022;19(3):1–25. 10.1371/journal.pmed.1003959 PMC8982898 35316272

[10] Zakumumpa H, Makobu K, Ntawiha W, Maniple E. A mixed-methods evaluation of the uptake of novel differentiated ART delivery models in a national sample of health facilities in Uganda. PLoS ONE. 2021;16(7):e0254214. 34292984 10.1371/journal.pone.0254214 PMC8297836

[11] Zakumumpa H, Rujumba J, Kwiringira J, Katureebe C, Spicer N. Understanding implementation barriers in the national scale-up of differentiated ART delivery in Uganda. 2020;1–16. 10.1186/s12913-020-5069-y PMC7077133 32183796

[12] Kintu TM, Ssewanyana AM, Kyagambiddwa T, Nampijja PM, Apio PK, Kitaka J, et al. Exploring drivers and barriers to the utilization of community client-led ART delivery model in South-Western Uganda: patients’ and health workers’ experiences. BMC Health Serv Res. 2021;21:1129. 34670564 10.1186/s12913-021-07105-9 PMC8527820

[13] Huber A, Pascoe S, Nichols B, Long L, Kuchukhidze S, Phiri B, et al. Differentiated Service Delivery models for HIV Treatment in Malawi, South Africa, and Zambia: A Landscape Analysis. Glob Health Sci Pract. 2021;9(2):296–307. 34234023 10.9745/GHSP-D-20-00532 PMC8324204

[14] USAID PEPFAR, MEASURE Evaluation. A Case Study to Measure National HIV M&E System Strengthening Nigeria. 2014.

[15] Ehrenkranz P, Grimsrud A, Holmes CB, Preko P, Rabkin M. Expanding the Vision for Differentiated Service Delivery: A Call for More Inclusive and Truly Patient-Centered Care for People Living With HIV. J Acquir Immune Defic Syndr (1988). 2021;86(2):147–52. 10.1097/QAI.0000000000002549 PMC7803437 33136818

[16] The Republic of Uganda. The state of Uganda Population Report 2022. Nov: Kampala; 2022.

[17] The Republic of Uganda. UPHIA-Summary-Sheet-2020. Uganda Population-based HIV Impact Assessment- UPHIA 2020–2021. Kampala; 2022. pp. 1–3.

[18] Bradbury-Jones C, Sambrook S, Irvine F. The phenomenological focus group: an oxymoron? J Adv Nurs. 2009;65(3):663–71. 19222664 10.1111/j.1365-2648.2008.04922.x

[19] Thomas DR. A general inductive approach for qualitative data analysis. 2003.

[20] Tong A, Sainsbury P, Craig J. Consolidated criteria for reporting qualitative research (COREQ): a 32-item checklist for interviews and focus groups. Int J Qual Health Care. 2007;19(6):349–57. 17872937 10.1093/intqhc/mzm042

[21] Limbada M, Macleod D, Situmbeko V, Muhau E, Shibwela O, Chiti B, et al. Rates of viral suppression in a cohort of people with stable HIV from two community models of ART delivery versus facility-based HIV care in Lusaka, Zambia: a cluster-randomised, non-inferiority trial nested in the HPTN 071 (PopART) trial. Lancet HIV. 2022;9(1):e13–23. 34843674 10.1016/S2352-3018(21)00242-3 PMC8716341

[22] Baleeta K, Muhwezi A, Tumwesigye N, Kintu BN, Riese S, Byonanebye D, et al. Factors that influence the satisfaction of people living with HIV with differentiated antiretroviral therapy delivery models in east central Uganda: a cross-sectional study. BMC Health Serv Res. 2023;23(1):1–11. 36750840 10.1186/s12913-023-09114-2 PMC9906920

[23] Mukumbang FC, Ndlovu S, van Wyk B. Comparing patients’ experiences in three differentiated Service Delivery models for HIV Treatment in South Africa. Qual Health Res. 2022;32(2):238–54. 34911400 10.1177/10497323211050371 PMC8727825

[24] Christ B, van Dijk JH, Nyandoro TY, Reichmuth ML, Kunzekwenyika C, Chammartin F, et al. Availability and experiences of differentiated antiretroviral therapy delivery at HIV care facilities in rural Zimbabwe: a mixed-method study. J Int AIDS Soc. 2022;25(8):1–12. 10.1002/jia2.25944 PMC9411726 36008925

[25] Nkolo EKK, Ejike JC, Sensalire S, Ssali JN, Ddumba I, Calnan J, et al. Clients in Uganda accessing preferred differentiated antiretroviral therapy models achieve higher viral suppression and are less likely to miss appointments: a cross-sectional analysis. J Int AIDS Soc. 2023;26:e26122. 37408483 10.1002/jia2.26122 PMC10323312

[26] Mukumbang FC. Leaving no man behind: how differentiated service delivery models increase men’s engagement in HIV care. Int J Health Policy Manag. 2021;10(3):129–40. 32610748 10.34172/ijhpm.2020.32 PMC7947905

[27] Nkolo EKK, Jessica Clinkscales Ejike, Sensalire S, Ssali JN, Ddumba I, Calnan J, et al. Clients in Uganda accessing preferred differentiated antiretroviral therapy models achieve higher viral suppression and are less likely to miss appointments: a cross-sectional analysis. J Int AIDS Soc. 2023;26(S1):31–8. 10.1002/jia2.26122 PMC10323312 37408483

[28] Mody A, Roy M, Sikombe K, Savory T, Holmes C, Bolton-Moore C, et al. Improved Retention with 6-Month Clinic Return intervals for stable human immunodeficiency virus-infected patients in Zambia. Clin Infect Dis. 2018;66(2):237–43. 29020295 10.1093/cid/cix756 PMC5850531

[29] Okere NE, Meta J, Maokola W, Martelli G, van Praag E, Naniche D, et al. Quality of care in a differentiated HIV service delivery intervention in Tanzania: a mixed-methods study. PLoS ONE. 2022;17(3 March):1–25. 10.1371/journal.pone.0265307 PMC8923447 35290989

[30] Okere NE, Urlings L, Naniche D, De Wit TFR, Gomez GB, Hermans S. Evaluating the sustainability of differentiated service delivery interventions for stable ART clients in sub-saharan Africa: a systematic review protocol. BMJ Open. 2020;10(1):1–7. 10.1136/bmjopen-2019-033156 PMC7045032 32014874

[31] Phillips AN, Bansi-Matharu L, Cambiano V, Ehrenkranz P, Serenata C, Venter F, et al. The potential role of long-acting injectable cabotegravir–rilpivirine in the treatment of HIV in sub-saharan Africa: a modelling analysis. Lancet Glob Health. 2021;9(5):e620–7. 33770513 10.1016/S2214-109X(21)00025-5 PMC8050198

[32] Kerrigan D, Mantsios A, Gorgolas M, Montes ML, Pulido F, Brinson C, et al. Experiences with long-acting injectable ART: a qualitative study among PLHIV participating in a phase II study of cabotegravir + rilpivirine (LATTE-2) in the United States and Spain. PLoS ONE. 2018;13(1):1–11. 10.1371/journal.pone.0190487 PMC5755771 29304154

[33] Jolayemi O, Bogart LM, Storholm ED, Goodman-Meza D, Rosenberg-Carlson E, Cohen R, et al. Perspectives on preparing for long-acting injectable treatment for HIV among consumer, clinical and nonclinical stakeholders: a qualitative study exploring the anticipated challenges and opportunities for implementation in Los Angeles County. PLoS ONE. 2022;17(2 February):1–21. 10.1371/journal.pone.0262926 PMC8812879 35113892

[34] UNAIDS. In Danger: UNAIDS Global AIDS Update 2022. Vol. CC BY-NC-S, World Heritage Review. Geneva; 2022.

