# Supplementary material for: Enhancing HIV treatment and support: a qualitative inquiry into client and healthcare provider perspectives on differential service delivery models in Uganda
Source: AIDS Res Ther. 2024 Jul 27;21:47. doi: 10.1186/s12981-024-00637-0 (PMC11282821; doi:10.1186/s12981-024-00637-0)
Supplement: Supplementary file 1 — Supplementary Material 1 [file 12981_2024_637_MOESM1_ESM.doc]

**T6 – In-depth Interview (IDI) or FGD with Caregivers in the Care Giver-DOTS Model**

Note: This tool may be used for a IDI or FGD depending on the number of Caregivers

| 1. Intro question:    1. What is your relationship with the child or children you are giving care to?    2. What interested you to get involved in giving care for the child or children you care for?    3. What activities to you as part of the care you provide? What did you like most about these activities? |
| --- |
| 1. Have you observed changes regarding the children’s behaviours regarding HIV treatment? Please provide examples.    1. What are the changes you have observed?    2. How different was the situation before you started given care under the Caregiver DOTs model?    3. Have you observed any changes regarding the child or children’s keeping of ART appointment, adherence to ART, and/or disclosure of HIV status? Please give examples.    4. How different was the situation of keeping ART appointment, adherence to ART and disclosure of HIV status before the Care Giver DOTS model? |
| 1. Related to the above questions in 2.1 and 2.3, which particular activities do you think helped achieve these changes?    1. Are there specific situations where the activities in the Caregiver DOTS worked better e.g. in other sub-counties or parishes, urban vs rural?    2. What were supporting factors that helped make the activities successful?    3. Do you think the observed (positive) changes that you have observed because of the Caregiver DOTS will continue after Mildmay Uganda and the partner stop their support to the district or health facility? If yes, explain. If no, why?    4. Were there any unintended negative effects of the Caregiver DOTS so far? In your opinion, what can be done to address or prevent these negative effects? |
| 1. To your knowledge, does the Caregiver DOTS model involve vulnerable children (for example, children with disabilities, orphans etc)? Please share examples.    1. How have they benefited from the programme?    2. How do you think they can be much more involved/included? |
| - 1. What are the challenges that affect the children’s care and treatment?   2. What challenges do you face as a caregiver with regard to the Caregiver DOTs services?   3. How can these chanllenges be addressed? |
| 1. Do you have suggestions or recommendations to increase the impact (or positive results) of the Caregiver DOTS in your district? |
