# Supplementary material for: Enhancing HIV treatment and support: a qualitative inquiry into client and healthcare provider perspectives on differential service delivery models in Uganda
Source: AIDS Res Ther. 2024 Jul 27;21:47. doi: 10.1186/s12981-024-00637-0 (PMC11282821; doi:10.1186/s12981-024-00637-0)
Supplement: Supplementary file 2 — Supplementary Material 2 [file 12981_2024_637_MOESM2_ESM.docx]

## T4 – IDI or FGD Tool for Differential Service Delivery Models

## District: ______________________________________________ Sub-county: ____________________________________________

## Health Facility: _______________________________________ Model(s): ___________________________________________________

**Note:** This tool may be used for a IDI or FGD depending on the number of target groups, implementation context, locations, etc.

| 1. Intro question  1.1 Which differential service delivery model(s) (DSDM) are you enrolled in?  1.2 Please share examples of activities you were involved in as part of the DSDM. |
| --- |
| 1. In your opinion, what are the biggest challenges to keeping ART appointments, and adhering to ART in your district/community?    1. In your view, how has the DSDM you are enrolled in addressed these challenges in your district/community? |
| 3. Have you observed changes in members of your DSDM regarding their keeping of ART appointments, adhering to treatment, disclosure of HIV status, falling ill from opportunistic infections etc?  3.1. What are the changes you have observed among members of your DSDM, if any?  3.2. How different was the situation of keeping of ART appointments, adhering to treatment, disclosure of HIV status, falling ill from opportunistic infections etc before joining the DSDM?  3.3 Are there specific things that the DSDM has done that has led to these changes? Please give examples.  3.4 Do you think these changes will continue after Mildmay Uganda and its partner(s) stop supporting the district/health facility? Please give reason for your answer. |
| 4. In your view, how has enrolling in your DSDM affected the following:  4.1. Accessibility to ART services?  4.2 Convenience/flexibility of the time/days etc of picking up drugs?  4.3 Stigma associated with picking up ART medicines from health facility?  4.4 How much time you or others spent when picking ARVs from health facility?  4.5 Issues of congestion and risk of acquiring other infections such as TB, COVID-19?  4.6 Access to other medicines for other conditions/health problems apart from HIV? |
| 5.1 What challenges do clients enrolled in your DSDM face while on ART?  5.2 How can these challenges be addressed? |
| 6 Do you have suggestions or recommendations to increase the impact (or positive results) of the DSDM you are enrolled in? |
