# Supplementary material for: Enhancing HIV treatment and support: a qualitative inquiry into client and healthcare provider perspectives on differential service delivery models in Uganda
Source: AIDS Res Ther. 2024 Jul 27;21:47. doi: 10.1186/s12981-024-00637-0 (PMC11282821; doi:10.1186/s12981-024-00637-0)
Supplement: Supplementary file 4 — Supplementary Material 4 [file 12981_2024_637_MOESM4_ESM.docx]

## T5 – IDI Tool for Differential Service Delivery Models

## District: ______________________________________________ Sub-county: ____________________________________________

## Health Facility: _______________________________________ Model(s): ___________________________________________________

**Note:** This tool should be used for Key Informant Interview (KII) with service providers, including pharmacy operators for the Community Retail Pharmacy

| 1. Intro question  1.1 Which differential service delivery model(s) (DSDM) do you or does your facility or district have?  1.2 Please share examples of activities you, the health facility or district carry in the different DSDM, YAPS and Care-Giver Led DOTS. |
| --- |
| 1. In your opinion, what are the biggest challenges clients face in keeping ART appointments, and adhering to ART in your district/health facility/community?   2.1 What challenges do service providers face trying to ensure clients follow appointment and adhere to ART?  2.2 In your view, how has implementation of the different DSDMs, YAPS and caregiver DOTS addressed these challenges in your district/health facility/community? |
| 3. Have you observed changes in members of the different DSDM, YAPS and caregiver-DOTS regarding their keeping of ART appointments, adhering to treatment, disclosure of HIV status, falling ill from opportunistic infections etc? (Please probe on each DSDM, YAPS or caregiver-DOTS independently)  3.1. What are the changes you have observed?  3.2 Which DSDM do you think has brought the most changes? Why do you think or say so?  3.3. How different was the situation of keeping of ART appointments, adhering to treatment, disclosure of HIV status, falling ill from opportunistic infections etc different before the roll out of DSDMs?  3.4 Are there specific things that the DSDM has done that has led to these changes? Please give examples.  3.4 Do you think these changes will continue after Mildmay Uganda and its partner(s) stop supporting the district/health facility? Please give reason for your answer. |
| 4. In your view, how have the different DSDM, YAPS, caregiver-DOTS affected the following:  4.1. Accessibility to ART services?  4.2 Convenience/flexibility of the time/days etc of picking up drugs?  4.3 Stigma associated with picking up ART medicines from health facility?  4.4 How much time you or others spent when picking ARVs from health facility?  4.5 Issues of congestion and risk of acquiring other infections such as TB, COVID-19?  4.6 Access to other medicines for other conditions/health problems apart from HIV? |
| 5 Do you have suggestions or recommendations to increase the impact (or positive results) of the DSDM you are enrolled in? |
