# Supplementary material for: Enhancing HIV treatment and support: a qualitative inquiry into client and healthcare provider perspectives on differential service delivery models in Uganda
Source: AIDS Res Ther. 2024 Jul 27;21:47. doi: 10.1186/s12981-024-00637-0 (PMC11282821; doi:10.1186/s12981-024-00637-0)
Supplement: Supplementary file 5 — Supplementary Material 5 [file 12981_2024_637_MOESM5_ESM.docx]

**YAPS FGD Tool with Young People and Adolescent Peer Support (YAPS) Groups**

Note: This tool may be used for a IDI or FGD depending on the number of young people, implementation context, locations, etc.

| 1. Intro question:    1. What interested you in getting involved with the YAPS?    2. Please share examples of activities you were involved in as part of the YAPS group. What did you like most about these activities?    3. Were there actions that you or your fellow youth did after participating in YAPS group meetings, health education sessions, peer support etc.? Please share examples. |
| --- |
| 1. Have you observed changes in your own or your peers (i.e. other members of the YAPS group) regarding their views, knowledge or behaviours regarding HIV prevention and treatment? Please provide examples.    1. What are the changes you have observed in your district/communities, if any?    2. How different was the situation of your peers’ views, knowledge or behaviours before joining the YAPS group?    3. Have you observed any changes regarding your peers’ keeping of ART appointment, adherence to ART, and disclosure of HIV status? Please give examples.    4. How different was the situation of keeping ART appointment, adherence to ART and disclosure of HIV status before joining the YAPS group? |
| 1. Related to the above questions in 2.1 and 2.3, which particular activities do you think helped achieve these changes?    1. Are there specific situations where the activities in the YAPS groups worked better e.g. in other sub-counties or parishes, urban vs rural?    2. What were supporting factors that helped make the activities successful?    3. Do you think the observed (positive) changes that you have observed because of the YAPS will continue after Mildmay Uganda and the partner stop their support to the district or youth groups? If yes, explain. If no, why?    4. Were there any unintended negative effects of the YAPS groups so far? In your opinion, what can be done to address or prevent these negative effects? |
| 1. To your knowledge, did the groups involve young people that are usually excluded or disadvantaged (for example, girls, persons with disabilities, and other marginalised young people)? Please share examples.    1. How have they benefited from the programme?    2. How do you think they can be much more involved/included? |
| - 1. What are the challenges that affect the adolescents in carrying out their support activities in the YAPS groups?   2. How can these chanllenges be addressed? |
| 1. Do you have suggestions or recommendations to increase the impact (or positive results) of the YAPS groups in your district? |
